# Supplementary material for: Community-Based Mycetoma Surveillance in Uganda: Identifying Knowledge Gaps and Training of Community Health Workers to Improve Case Detection
Source: PLoS Negl Trop Dis. 2024 Oct 7;18(10):e0012572. doi: 10.1371/journal.pntd.0012572 (PMC11486432; doi:10.1371/journal.pntd.0012572)
Supplement: S2 Data — (DOCX) [file pntd.0012572.s002.docx]

STROBE Checklist

|  | Item No | Recommendation |
| --- | --- | --- |
| **Title and abstract** | 1 | (*a*) Indicate the study’s design with a commonly used term in the title or the abstract-Page 1 |
|  |  | (*b*) Provide in the abstract an informative and balanced summary of what was done and what was found-Page 2 |
| Introduction | | |
| Background/rationale | 2 | Explain the scientific background and rationale for the investigation being reported-Page 4,5 |
| Objectives | 3 | State specific objectives, including any prespecified hypotheses-Page 6 |
| Methods | | |
| Study design | 4 | Present key elements of study design early in the paper-Page 6 |
| Setting | 5 | Describe the setting, locations, and relevant dates, including periods of recruitment, exposure, follow-up, and data collection-Page 6, 7 |
| Participants | 6 | (*a*) Give the eligibility criteria, and the sources and methods of selection of participants-Page 6 |
| Variables | 7 | Clearly define all outcomes, exposures, predictors, potential confounders, and effect modifiers. Give diagnostic criteria, if applicable-Page 7 |
| Data sources/ measurement | 8* | For each variable of interest, give sources of data and details of methods of assessment (measurement). Describe comparability of assessment methods if there is more than one group-Page 7 |
| Bias | 9 | Describe any efforts to address potential sources of bias-Page 7 |
| Study size | 10 | Explain how the study size was arrived at- page 6 |
| Quantitative variables | 11 | Explain how quantitative variables were handled in the analyses. If applicable, describe which groupings were chosen and why-Page 7 |
| Statistical methods | 12 | (*a*) Describe all statistical methods, including those used to control for confounding-Page 9 |
|  |  | (*b*) Describe any methods used to examine subgroups and interactions-Page 9 |
|  |  | (*c*) Explain how missing data were addressed-Page 9 |
|  |  | (*d*) If applicable, describe analytical methods taking account of sampling strategy-Page 9 |
|  |  | (*e*) Describe any sensitivity analyses-Page 9 |
| Results | | |
| Participants | 13* | (a) Report numbers of individuals at each stage of study—eg numbers potentially eligible, examined for eligibility, confirmed eligible, included in the study, completing follow-up, and analysed-Page 10 |
| Descriptive data | 14* | (a) Give characteristics of study participants (eg demographic, clinical, social) and information on exposures and potential confounders-Page 10 |
|  |  | (b) Indicate number of participants with missing data for each variable of interest-Page 10 |
| Outcome data | 15* | Report numbers of outcome events or summary measures-Page 12 |
| Main results | 16 | (*a*) Give unadjusted estimates and, if applicable, confounder-adjusted estimates and their precision (eg, 95% confidence interval). Make clear which confounders were adjusted for and why they were included-Page 12 |
|  |  |  |
|  |  |  |
| Other analyses | 17 | Report other analyses done—eg analyses of subgroups and interactions, and sensitivity analyses-Page 12,13 |
| Discussion | | |
| Key results | 18 | Summarise key results with reference to study objectives-page 18 |
| Limitations | 19 | Discuss limitations of the study, taking into account sources of potential bias or imprecision. Discuss both direction and magnitude of any potential bias-Page 19 |
| Interpretation | 20 | Give a cautious overall interpretation of results considering objectives, limitations, multiplicity of analyses, results from similar studies, and other relevant evidence-Page 18, 19 |
| Generalisability | 21 | Discuss the generalisability (external validity) of the study results-Page 19 |
| Other information | | |
| Funding | 22 | Give the source of funding and the role of the funders for the present study and, if applicable, for the original study on which the present article is based-Page 20 |
