# Supplementary material for: Community-Based Mycetoma Surveillance in Uganda: Identifying Knowledge Gaps and Training of Community Health Workers to Improve Case Detection
Source: PLoS Negl Trop Dis. 2024 Oct 7;18(10):e0012572. doi: 10.1371/journal.pntd.0012572 (PMC11486432; doi:10.1371/journal.pntd.0012572)
Supplement: Attachment — Submitted filename: Uganda PLOS review.docx [file pntd.0012572.s005.docx]

**Task: Review of Paper**

**Manuscript Number:** PNTD-D-24-00746 for reviewing the paper

**Title:**

Community-Based Mycetoma Surveillance in Uganda: Identifying Knowledge Gaps and Training of Community Health Workers to Improve Case Detection.

**Remarks:**

1. The topic deserves to be investigated, based on the stated need (Lines 43-46). This research is linked to some observed gaps in case of mycetoma detection among tropical communities. It aimed to see the impacts of training of community health workers ‘CHWs’. It aspired to augment some community/field practices for early detection of the lesser known cases of mycetoma in Uganda. So, this research is justified in concept.

2. The Abstract and the author summary are agreeing with the overall objective of the work (Lines 22-64).

3. At starting the introduction, the causal agent/s of mycetoma.is generally defined, It could be better to highlight the causal agent/s, with their relevant scientific terms (Line 65). For example, Madurella mycetomatis was the most prevalent causative agent world-wide, followed by Actinomadura madurae, Streptomyces somaliensis, Actinomadura pelletieri,  Nocardia brasiliensis and Nocardia asteroides.

4. Researchers noted in a recent systematic review of the global burden of human mycetoma estimated the average number of reported cases at about 127 cases per annum, globally, as cited in reference 1 in the present paper under review. This is global case is rather under estimated and this figure of 127 cases needs to be revised, if considered for global level. It is probably per 1000 of inhabitants, For example, in the same cited reference 1, in the attached references, it is quoted ‘in Sudan, during 2012 only, 5158 patients were seen of which 402 were new cases. This means that there were (5158 cases/year) seen in Sudan only’. However, global cases needed to be re-visited.

5. **Methods section:**

**Study design and settings (Lines** 119-123):

A concurrent triangulation mixed-methods study among CHWs were used to assess barriers and facilitators to early detection and referral of cases of mycetoma in Pader and Gulu districts in Northern Uganda. Such statement needs to be qualified with the reference published method, if depending on a known publication. Otherwise, such combined methods should be briefly described in the present text.

**Data collection:**

With pre-and post-training assessment and intervention part is quite logical and well written. However, researchers purposely selected 45 CHWs operating within the catchment areas, Is this number of 45 CHWs is statistically validated choice or option, out of over 300 CHWs? In other words, did researchers tried any method for statistical validation while selecting/picking an optimum number of these CHWs? If yes, it could be noted in the text in order to strengthen the population choice case of the present study.

**Qualitative data:**

Data sets were collected, while examining the knowledge and awareness of CHWs on mycetoma. This included barriers and facilitators to early diagnosis and referral of suspected cases of mycetoma, as well as training needs, in order to support and enhance early detection and referral of mycetoma cases among many rural communities.

**Data Analysis:**

It is reported that quantitative data (Lines 184-185) were analyzed using GraphPad Prism 8.03. and STATA version 17.0. It will be clearer if quantitative tools of are indicated, or related to the respective set of data.

Researchers mentioned that knowledge scores were calculated out of a total score of 18, and bivariate comparisons were made using paired sample t-test or one-way Analysis of Variance (ANOVA) as appropriate (Line 186-188). In this analytical part, the text will be better explained and more understood, if indicating the basis for opting to either of paired sample t-test or one-way or ANOVA.

**Qualitative analysis:**

Thematic grouping (Lines 190-193) of the study population is quite convenient and well presented. The results (245-345) are also well illustrated, where other researchers often do not use such methods, despite their expressive value in explaining results of such investigation. The reviewer commends on this thematic use in research and presentation

The highlighted * in Table 3 below should worth revising the level of significance in the cases in red (Line 233).

**Table 3: Mean knowledge scores**

| Variable | Baseline  (Pre-Test) | 6 months  (Post-Test) | P-value |
| --- | --- | --- | --- |
| Sex |  |  |  |
| Female | 5.4 (3.23) | 11.2 (2.62) | 0.004 |
| Male | 8.3 (2.14) | 9.6 (2.03) | 0.060* |
| Age |  |  |  |
| <30 | 7.7(2.42) | 14.0(1.00) | 0.298* |
| 30-40 | 6.7(2.65) | 9.7(1.82) | 0.031 |
| >40 | 6.8(2.18) | 9.8(1.82) | 0.035 |
| Highest level of education |  |  |  |
| Primary | 6.0(1.7) | 11.0(-) | - |
| Secondary | 7.3(2.39) | 9.9(2.51) | 0.007 |
| Certificate | 6.6(3.26.) | 10.5(4.95) | 0.083* |
| Number of years of practice median (interquartile range) |  |  |  |
| <6 | 6.8(2.04) | 9.3(1.7) | 0.157* |
| 6-10 | 7.3(2.42) | 11.0(2.98) | 0.080* |
| >10 | 7.2(2.98) | 10.2(2.21) | 0.025 |

- P-value is more than 0.05

**Discussion and Conclusion:**

Both titles in this research are well covered. This research showed the significance of, as less as one day, training can make a positive impact and enhance level of detection in cases of mycetoma infections among rural communities.

**Final Conclusion:**

This type of exercise with CHWs could be applied for detection of other neglected tropical diseases. The outcome might be much better, if training is extended over 5-6 days, assisted with prepared manual/s for teaching trainees or CHWs in packages, directed to each of theses neglected tropical diseases. Such investment in CHWs might save communities with limited or meagre budget for rough diagnosis of a tropical neglected disease such as that of mycetoma. This method could be very valuable for the African healthcare systems in remote areas for diagnosis of similar neglected tropical diseases.
