# Supplementary material for: Community-Based Mycetoma Surveillance in Uganda: Identifying Knowledge Gaps and Training of Community Health Workers to Improve Case Detection
Source: PLoS Negl Trop Dis. 2024 Oct 7;18(10):e0012572. doi: 10.1371/journal.pntd.0012572 (PMC11486432; doi:10.1371/journal.pntd.0012572)
Supplement: Attachment — Submitted filename: RESPONSE TO REVIEWERS.docx [file pntd.0012572.s006.docx]

**RESPONSE TO REVIEWERS**

| **N0** | Comment | **Response** | **Page N0** |
| --- | --- | --- | --- |
| **Reviewer 1** | | |  |
| 1 | The research concept is robust, addressing the crucial issue of early detection of mycetoma to improve management and outcomes in remote rural communities. The study examines the knowledge of community health workers on the front lines, assessing their ability to identify this neglected disease | Thank you for your positive feedback on the research concept. | - |
| 2 | It should be explicitly stated in the manuscript that these workers do not aim to diagnose mycetoma but rather to detect suspected cases for referral to appropriate clinical, laboratory, and imaging diagnostic facilities. | Thank you. We agree with the reviewer. We have revised the text to explicitly reflect that the primary objective of community health workers is not to diagnose mycetoma but to identify suspected cases for referral to appropriate clinical, laboratory, and imaging diagnostic facilities. | Page 6 |
| 3 | Out of the 300 suspected patients, it is essential to clarify their subsequent outcomes, especially from an ethical standpoint. | Thank you for raising this important point. We would like to clarify that our study reports on 30 suspected cases, not 300. We acknowledge that a limitation of our study is that while the CHWs referred these patients to appropriate health facilities, we did not conduct follow-up with the facilities to ascertain the patients' outcomes. We have now included this as a limitation in the manuscript and have discussed the potential implications. We agree that understanding these outcomes is crucial, and future studies should incorporate a follow-up mechanism to address this gap. | Page 19 |
| 4 | If confirmed as mycetoma, this cohort would significantly contribute to understanding the prevalence of the disease in Uganda. | We agree that confirmation of these cases would provide valuable insights into the disease burden. However, due to the limitations in follow-up and diagnostic confirmation, we were unable to determine how many of the suspected cases were definitively diagnosed as mycetoma. We have acknowledged this as a limitation in the study. | Page 19 |
| 5 | The objectives and methodology of the study were clearly articulated.    •    Ethical considerations were thoroughly addressed.    •    Additional statistical analysis could be performed on the collected data to enhance the study’s findings. | Thank you for your positive feedback. While we conducted the necessary analyses to address our primary research questions, we agree that further analysis could provide additional insights. We will explore the feasibility of performing these additional analyses and consider incorporating them into subsequent studies. | Page 9 and 10 |
| 6 | The discussion section is relatively weak, with numerous repetitions from the results section. | Thank you for your feedback. We have revised the discussion to reduce redundancy. | Page 18 and 19 |
| 7 | Photos on the methodology will be helpful | Thank you for this suggestion. Unfortunately, we are not in position to include an image to represent the methodology. We have however described the methodology in detail. | Page 6-8 |
| 8 | Overall, the manuscript requires substantial revision to improve the quality of English and correct typographical errors. I have highlighted several statements in yellow with comments directly on the manuscript. | Thank you. We appreciate the time you have taken to provide comments directly on the text. We will carefully review each highlighted section and address the suggested revisions to improve the quality of English and correct typographical errors. |  |
| **Reviewer 2** | | |  |
|  |  |  |  |
| **Reviewer 3** | | |  |
| 1 | The topic deserves to be investigated, based on the stated need (Lines 43-46). This research is linked to some observed gaps in case of mycetoma detection among tropical communities. It aimed to see the impacts of training of community health workers ‘CHWs’. It aspired to augment some community/field practices for early detection of the lesser known cases of mycetoma in Uganda. So, this research is justified in concept. | Thank you for your positive feedback on the significance and justification of our research. |  |
| 2 | The Abstract and the author summary are agreeing with the overall objective of the work (Lines 22-64). | Thank you for recognizing the alignment between the Abstract, Author Summary, and the overall objective of our work. | Page 3 |
| 3 | At starting the introduction, the causal agent/s of mycetoma.is generally defined, It could be better to highlight the causal agent/s, with their relevant scientific terms (Line 65). For example, Madurella mycetomatis was the most prevalent causative agent world-wide, followed by Actinomadura madurae, Streptomyces somaliensis, Actinomadura pelletieri,  Nocardia brasiliensis and Nocardia asteroides. | Thank you. We have revised the introduction to include a more detailed description of the primary causative agents, including Madurella mycetomatis, Actinomadura madurae, Streptomyces somaliensis, Actinomadura pelletieri, Nocardia brasiliensis, and Nocardia asteroides, as recommended. | Page 4 |
| 4 | Researchers noted in a recent systematic review of the global burden of human mycetoma estimated the average number of reported cases at about 127 cases per annum, globally, as cited in reference 1 in the present paper under review. This is global case is rather under estimated and this figure of 127 cases needs to be revised, if considered for global level. It is probably per 1000 of inhabitants, For example, in the same cited reference 1, in the attached references, it is quoted ‘in Sudan, during 2012 only, 5158 patients were seen of which 402 were new cases. This means that there were (5158 cases/year) seen in Sudan only’. However, global cases needed to be re-visited. | Thank you. We agree with the reviewer. We have revised the manuscript to clarify this, including the possibility that the figures represent a significant underestimation. | Page 4 |
| 5 | **Methods section:**  **Study design and settings (Lines** 119-123):  A concurrent triangulation mixed-methods study among CHWs were used to assess barriers and facilitators to early detection and referral of cases of mycetoma in Pader and Gulu districts in Northern Uganda. Such statement needs to be qualified with the reference published method, if depending on a known publication. Otherwise, such combined methods should be briefly described in the present text. | Thank you. We have added a reference to support our study design. Additionally, we have briefly described the rationale behind using this method in our study. | Page 6 |
| 6 | With pre-and post-training assessment and intervention part is quite logical and well written. However, researchers purposely selected 45 CHWs operating within the catchment areas, Is this number of 45 CHWs is statistically validated choice or option, out of over 300 CHWs? In other words, did researchers tried any method for statistical validation while selecting/picking an optimum number of these CHWs? If yes, it could be noted in the text in order to strengthen the population choice case of the present study. | Thank you. We acknowledge the importance of statistical validation in determining sample size. In this pilot study, the selection of 45 CHWs was based on purposive sampling rather than statistical validation, primarily due to logistical constraints and the specific focus on CHWs operating within the catchment areas of Awach Health Centre IV in Gulu and Pajule Health Centre IV in Pader district. The aim was to gather initial insights and assess the feasibility of our intervention within a manageable cohort before scaling up the study to include a larger, statistically validated sample size. We have noted this limitation in our study and appreciate your suggestion to explicitly mention it in the manuscript to strengthen the rationale behind our sample selection. | Page 7 and 20 |
| 7 | **Qualitative data:**  Data sets were collected, while examining the knowledge and awareness of CHWs on mycetoma. This included barriers and facilitators to early diagnosis and referral of suspected cases of mycetoma, as well as training needs, in order to support and enhance early detection and referral of mycetoma cases among many rural communities. | Thank you. The statement in the manuscript has been edited. | Page 7 |
| 8 | **Data Analysis:**  It is reported that quantitative data (Lines 184-185) were analyzed using GraphPad Prism 8.03. and STATA version 17.0. It will be clearer if quantitative tools of are indicated, or related to the respective set of data.  Researchers mentioned that knowledge scores were calculated out of a total score of 18, and bivariate comparisons were made using paired sample t-test or one-way Analysis of Variance (ANOVA) as appropriate (Line 186-188). In this analytical part, the text will be better explained and more understood, if indicating the basis for opting to either of paired sample t-test or one-way or ANOVA. | Thank you for the feedback. This has been revised in the manuscript. | Page 9 |
| 9 | **Qualitative analysis:**  Thematic grouping (Lines 190-193) of the study population is quite convenient and well presented. The results (245-345) are also well illustrated, where other researchers often do not use such methods, despite their expressive value in explaining results of such investigation. The reviewer commends on this thematic use in research and presentation | Thank you. We appreciate your commendation of the use of thematic analysis in our study. | Page 9 and 20 |
| 10 | The highlighted * in Table 3 below should worth revising the level of significance in the cases in red (Line 233). **Table 3: Mean knowledge scores** Variable \| Baseline (Pre-Test) \| 6 months (Post-Test) \| P-value; Sex \| \| \|; Female \| 5.4 (3.23) \| 11.2 (2.62) \| 0.004; Male \| 8.3 (2.14) \| 9.6 (2.03) \| 0.060*; Age \| \| \|; <30 \| 7.7(2.42) \| 14.0(1.00) \| 0.298*; 30-40 \| 6.7(2.65) \| 9.7(1.82) \| 0.031; >40 \| 6.8(2.18) \| 9.8(1.82) \| 0.035; Highest level of education \| \| \|; Primary \| 6.0(1.7) \| 11.0(-) \| -; Secondary \| 7.3(2.39) \| 9.9(2.51) \| 0.007; Certificate \| 6.6(3.26.) \| 10.5(4.95) \| 0.083*; Number of years of practice median (interquartile range) \| \| \|; <6 \| 6.8(2.04) \| 9.3(1.7) \| 0.157*; 6-10 \| 7.3(2.42) \| 11.0(2.98) \| 0.080*; >10 \| 7.2(2.98) \| 10.2(2.21) \| 0.025 - P-value is more than 0.05 | Thank you for your comment. Unfortunately, the reviewer’s concern is not entirely clear. In this study, a p-value of less than 0.05 was considered statistically significant, as represented in the table 3. The values highlighted by the reviewer are not statistically significant. | Page 13 |
| 11 | **Discussion and Conclusion:**  Both titles in this research are well covered. This research showed the significance of, as less as one day, training can make a positive impact and enhance level of detection in cases of mycetoma infections among rural communities. | Thank you for your feedback on the discussion and conclusion sections of our research. | Page 18-20 |
| 12 | **Final Conclusion:**  This type of exercise with CHWs could be applied for detection of other neglected tropical diseases. The outcome might be much better, if training is extended over 5-6 days, assisted with prepared manual/s for teaching trainees or CHWs in packages, directed to each of theses neglected tropical diseases. Such investment in CHWs might save communities with limited or meagre budget for rough diagnosis of a tropical neglected disease such as that of mycetoma. This method could be very valuable for the African healthcare systems in remote areas for diagnosis of similar neglected tropical diseases. | We appreciate your thoughtful inputs, and agree that extending the training duration to 5-6 days could further enhance the effectiveness of community health workers in detecting a broader range of NTDs.  We will consider these valuable recommendations in future research and potential expansions of our training model. | Page 20 |
